# Synthesis and Antiscaling Evaluation of Novel Hydroxybisphosphonates for Oilfield Applications

**DOI:** 10.1021/acsomega.1c00379

**Published:** 2021-02-25

**Authors:** Mohamed F. Mady, Abdur Rehman, Malcolm A. Kelland

**Affiliations:** † Department of Chemistry, Bioscience and Environmental Engineering, Faculty of Science and Technology, University of Stavanger, N-4036 Stavanger, Norway; ‡ Department of Green Chemistry, National Research Centre, Dokki, Cairo 12622, Egypt

## Abstract

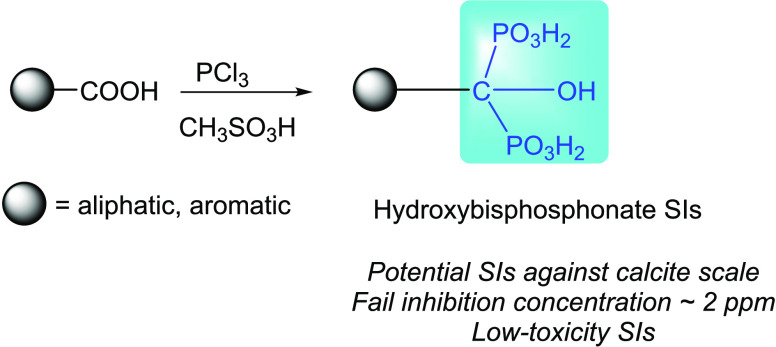

Organophosphorous compounds are still widely used as potential scale inhibitors in the upstream oil and gas industry, particularly in squeeze treatments as they have good adsorption properties on rock and are easily detectable. However, most phosphonate-based scale inhibitors have some drawbacks, such as poor biodegradability and various incompatibilities with the production system. The low toxicity of bisphosphonates motivated us to test a series of aliphatic and aromatic hydroxybisphosphonates as new oilfield scale inhibitors for calcium carbonate (calcite) and barium sulfate (barite) scales. Thus, the well-known bone-targeting drugs 3-amino-1-hydroxypropane-1,1-bisphosphonic acid (pamidronic acid, **SI-1**), 4-amino-1-hydroxybutane-1,1-bisphosphonic acid (alendronic acid, **SI-2**), 5-amino-1-hydroxypentane-1,1-bisphosphonic acid (**SI-3**), and hydroxyphenylmethylene-1,1-bisphosphonic acid (fenidronic acid, **SI-6**) are studied along with novel, specially designed bisphosphonates (1,4-dihydroxybutane-1,1,4,4-tetrayl)tetrakisphosphonic acid (**SI-4**), (1,6-dihydroxyhexane-1,1,6,6-tetrayl)tetrakisphosphonic acid (**SI-5**), and ((4- aminophenyl)(hydroxy)methylene)bisphosphonic acid (**SI-7**) in a dynamic tube-blocking scale rig at 100 °C and 80 bar according to typical North Sea conditions. The scale inhibition performance of the new SIs was compared to that of the commercial 1-hydroxyethylidene bisphosphonic acid (**HEDP**) and aminotrismethylenephosphonic acid (**ATMP**). The results indicate that all synthesized hydroxybisphosphonates provide reasonable inhibition performance against calcite scaling and show good thermal stability at 130 °C for 7 days under anaerobic conditions.

## Introduction

Inorganic scale formation is the precipitation of sparingly soluble inorganic salts from aqueous solutions.^1^ Oilfield scale is caused by deposition in the petroleum reservoir due to chemical incompatibility between well brines and injection waters.^2,3^ Mineral scales impact on fluid flow and hydrocarbon productivity by blocking production tubing, valves of a wellbore, and rock pores of the reservoir. Alongside corrosion and gas hydrates, scale deposition is also a most challenging problem during oil production and must be predicted in advance to avoid any severe loss.^4^ The most common mineral scales associated with oilfield applications are calcium carbonate (calcite) and sulfates of Group II metal ions such as barium (barite), calcium (gypsum), and strontium (celestite).^5,6^

Scale deposition can occur by two crystallization routes, which are bulk crystallization and surface crystallization.^7^ Other physical conditions such as pressure, pH, flow velocity, temperature, permeation rate, and coexistence of other ionizable particles also affect scale formation during operations. As stated earlier, the incompatibility among the anions and cations in two waters plays a primary role in scale formation. Therefore, adjusting the salinity of injection water has a significant role in preventing scale formation in production operations.^8^

A widely used method for oilfield scale management is using scale inhibitors (SIs). SIs are low-dosage water-soluble chemical additives that inhibit nucleation, crystal growth, and precipitation of mineral scales in the petroleum reservoir.^9,10^ Commonly used SIs are polymeric and/or nonpolymeric organic compounds incorporating scaling inhibition functional moieties such as phosphonate, carboxylate, and sulfonate groups.^11,12^ Phosphonate-based SIs have been deployed in the oil and gas industry for many years. Phosphonate SIs show an excellent scale inhibition performance for calcium carbonate and Group II sulfate scales under harsh conditions such as in high-pressure, high-temperature (HPHT) reservoirs.^13^ In addition, these classes of chemicals present superior binding to reservoir rocks, leading to prolonged squeeze lifetime treatment. However, they have some drawbacks, such as poor biodegradability properties and intolerance to high concentrations of calcium ions.^14^

Most commercial phosphonate SIs are associated with aminomethylenephosphonate groups. These inhibitors can be synthesized via the Moedritzer–Irani reaction, in which an amine derivative reacts with formaldehyde and phosphorous acid in the presence of hydrochloric acid.^15^ For example, aminotrismethylenephosphonic acid (**ATMP**), ethylenediamine tetra methylenephosphonic acid (**EDTMP**), diethylenetriaminepentamethylenephosphonic acid (**DTPM**P), hexamethylenediaminetetramethylenephosphonic acid (**HDTMP**), and bishexamethylenetriaminepentamethylenephosphonic acid (**BHMTMP**) are commonly used in the upstream oil and gas industry, particularly for squeeze treatments.^16^

**Figure 1:**
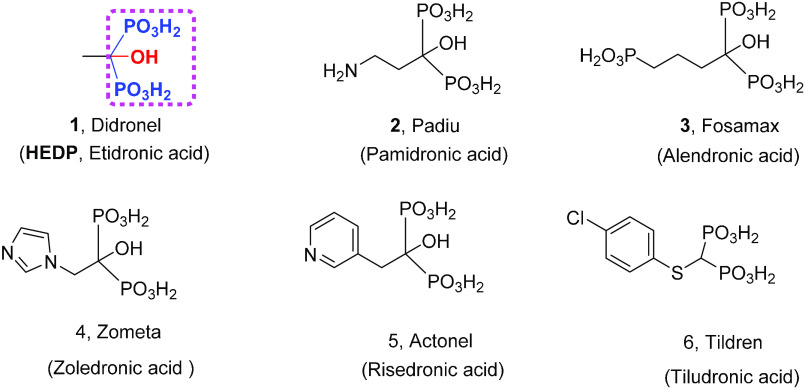
Chemical structures of commercial drugs containing bisphosphonate groups.

In addition to the classical aminomethylenephosphonate SIs, a few inhibitors based on bisphosphonate groups (PO_3_H_2_–C–PO_3_H_2_) have been used in the petroleum industry.^2,17,18^ Bisphosphonates (BPs) are biological analogues of naturally occurring components, pyrophosphates (P–O–P).^19^ BPs have widespread commercial acceptance for a variety of industrial and medical applications.^20−23^ Most BPs are well-known drugs in the treatment of osteoporosis and malignant bone diseases.^24,25^Figure 1 shows some of the commercial drugs based on BPs, which are clinically used to treat bone disorders.

Due to increasing environmental concern and discharge limitation of oilfield chemicals in the marine environment, several attempts to make more nontoxic and biodegradable SIs based on phosphonate groups have been reported.^26,27^ 1-Hydroxyethylidene bisphosphonic acid (**HEDP**, Figure 1) and its salts are widely used as scale and corrosion inhibitors in the oil and gas industry.^2,18^ Recently, Mady et al. synthesized a series of novel BPs based on amino groups showing antiscaling properties for calcite and barite scales, which revealed a moderate inhibition compared to the commercial products ATMP and DTPMP.^28^

**Figure 2:**
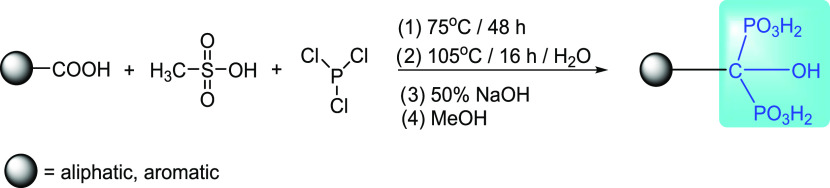
General scheme for the synthesis of hydroxybisphosphonates from acid derivatives.

According to the literature, several synthetic routes have been reported to synthesize hydroxybisphosphonates from the corresponding carboxylic acids in the presence of phosphorus trichloride and phosphorous acid.^29,30^ Grün et al. reported a green synthetic pathway for hydroxybisphosphonate derivatives using 3.2 equiv of phosphorus trichloride and methanesulfonic acid (MsOH) as a solvent.^31^Figure 2 shows the general procedure for the preparation of hydroxybisphosphonate derivatives.

In this work, the low toxicity of BPs motivated us to design and synthesize a series of hydroxybisphosphonate derivatives as scale inhibitors in the upstream oil and gas industry. For the first time, we report the calcite and barite scale inhibition performance for a series of well-known drugs used in the treatment of bone diseases, 3-amino-1- hydroxypropane-1,1-bisphosphonic acid (pamidronic acid, **SI-1**), 4-amino-1-hydroxybutane-1,1-bisphosphonic acid (alendronic acid, **SI-2**), 5-amino-1-hydroxypentane-1,1-bisphosphonic acid (**SI-3**), and hydroxyphenylmethylene-1,1-bisphosphonic acid (fenidronic acid, **SI-6**), as well as novel BPs (1,4-dihydroxybutane-1,1,4,4-tetrayl)tetrakisphosphonic acid (**SI-4**), (1,6-dihydroxyhexane-1,1,6,6-tetrayl)tetrakisphosphonic acid (**SI-5**), and ((4-aminophenyl)(hydroxy)methylene)bisphosphonic acid (**SI-7**). Aliphatic and aromatic hydroxybisphoshonates derived from biodegradable cores such as benzoic acid and β-alanine were prepared and screened for calcite and barite scale inhibition according to the Heidrun oilfield, North Sea, Norway. β-Alanine is a modified class of the amino acid alanine.^32^ The inhibition performance of the synthesized compounds was compared to that of a commercial BP-SI (**HEDP**), **ATMP**, and the laboratory sample of 1-aminoethylidene bisphosphonic acid (**AEDP**) using a high-pressure dynamic tube-blocking rig at approximately 80 bar and 100 °C. In addition, all synthesized inhibitors were evaluated for calcium compatibility and thermal stability.

## Experimental Section

### Chemicals

All chemicals and solvents used for synthesis were purchased from VWR, Nippon Chemical Industrial Co., Ltd.; Tokyo Chemical Industry Co., Ltd.; and Sigma-Aldrich (Merck). The sodium salt of 1-hydroxyethylidene bisphosphonic acid (**HEDP**) was obtained from Tokyo Chemical Industry Co., Ltd. 1-Aminoethylidene bisphosphonic acid (**AEDP**) was synthesized by reacting acetonitrile with phosphorous acid in the presence of phosphorus trichloride, as described in our previously published article.^28^

### Synthesis of Hydroxybisphosphonate Scale Inhibitors (SIs)

#### General Procedure for the Synthesis of Aliphatic and Aromatic Hydroxybisphosphonates from Carboxylic Acid Derivatives

A series of hydroxybisphosphonate SIs were synthesized based on phosphonation of carboxylic acid derivatives, as shown in Figure 2. In addition, Table 1 shows the chemical structures of all synthesized hydroxybisphosphonate SIs. All known hydroxybisphosphonates (**SI-1**, **SI-2**, **SI-3**, and **SI-6**), as well as three new BPs (**SI-4**, **SI-5**, and **SI-7**), were synthesized according to the general procedure reported by Grün et al.^31^ For example, (1,6-dihydroxyhexane-1,1,6,6-tetrayl)tetrakisphosphonic acid (**SI-5**) was synthesized by the reaction of adipic acid with phosphorus trichloride in methanesulfonic acid as follows:

**Table 1:**
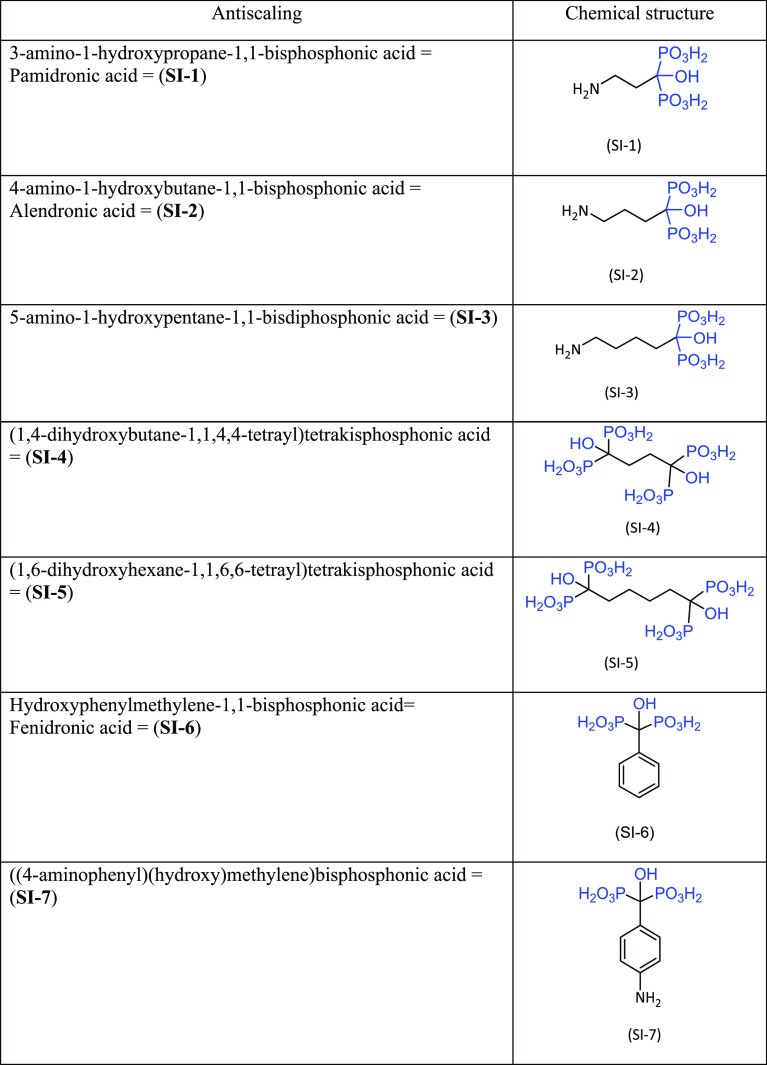
List of Aliphatic and Aromatic Hydroxybisphosphonates as Oilfield Scale Inhibitors

**Figure 3:**
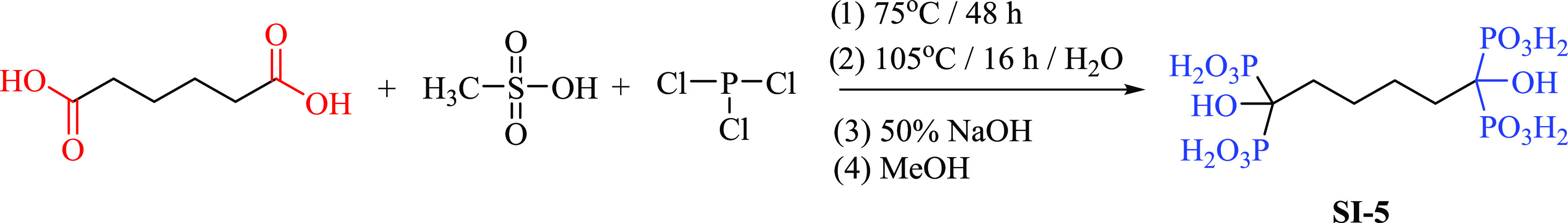
Synthesis of phosphonated adipic acid (**SI-5**).

In a two-neck round-bottom flask under nitrogen, 3.0 g (20.53 mmol) of adipic acid was added into 17.76 mL (184.81 mmol) of methanesulfonic acid under stirring at room temperature. Then, 18.04 g (131.36 mmol) of phosphorous trichloride was added dropwise for ca. 30 min and allowed to heat stepwise from room temperature to 75 °C. The reaction mixture was stirred for 48 h at 75 °C. After that, the mixture was cooled to room temperature and quenched carefully with 39.12 mL of deionized H_2_O. The reaction solution was then heated up and refluxed at 105 °C for 16 h. Next, the pH of the flask contents was adjusted to 1.8 by adding a 50% aqueous solution of NaOH, and then 127 mL of methanol was added into the mixture, followed by stirring for 45 min at room temperature. The crude product was collected by filtration and dissolved in 10 mL of hot water. Furthermore, 50 mL of methanol was added dropwise into the flask, providing an off-white solid. The phosphonated adipic acid (**SI-5**) was isolated as a pure product in 62% yield (Figure 3).

The structures of synthesized scale inhibitors were determined by nuclear magnetic resonance (NMR) spectroscopy. The NMR spectra were recorded on a 400 MHz Bruker NMR spectrometer in deuterium oxide (D_2_O) with two drops of sodium deuteroxide solution. ^1^H NMR and ^31^P NMR chemical shifts were recorded in D_2_O.

### High-Pressure Dynamic Tube-Blocking Test Methods

The performance of commercial and synthesized scale inhibitors was determined by a high-pressure dynamic tube-blocking test, as described previously by our research group.^33−37^ The obtained results from this test give a good assessment of the minimum inhibitor concentration (MIC) for SIs. SIs are often deployed in very low concentrations in the water medium. Therefore, an acceptable MIC value is in the range of 1–100 ppm, but our target is between 1 and 5 ppm. In this study, the test was used to evaluate the inhibition performance of the SI for calcite and barite oilfield scales. Dynamic tube-blocking tests to indicate the corresponding SI performance were performed on an automated scale rig (manufactured by Scaled Solutions Ltd., Scotland).

**Figure 4:**
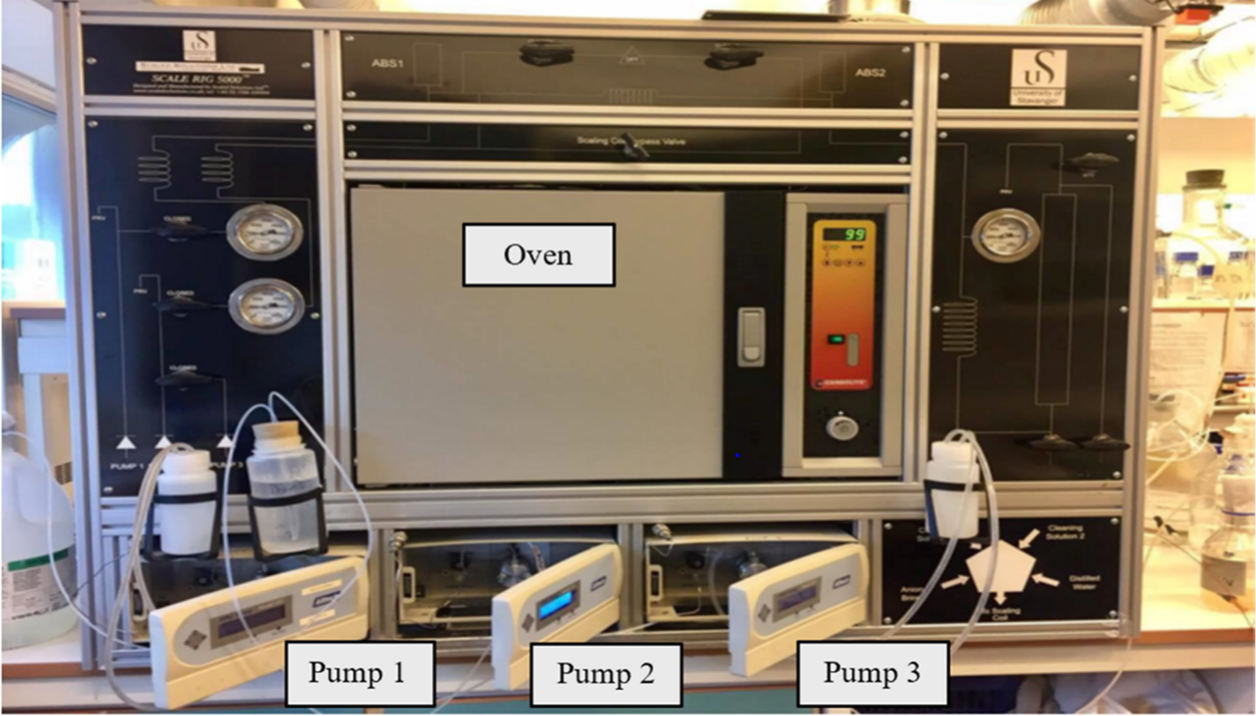
Schematic representation of the scale rig used for high-pressure tube-blocking testing of SIs.

There are three pumps in the scale rig that supply the specified fluids at up to 10.00 mL/min through a 3.00 m long microbore coil, as presented in Figure 4. The coil is made up of 316 steel with an internal diameter of 1 mm and located in an oven, which in this experiment was adjusted at 100 °C and the pressure in the coil was 80.0 bar. These pumps are labeled with numbers 1, 2, and 3. Pump 1 is to supply brine 1 (cationic solution), pump 2 is to supply brine 2 (anionic solution), and pump 3 is to supply a specified SI solution. Pump 2 is also used to inject the cleaning solution, consisting of 5 wt % tetrasodium ethylenediaminetetraacetate (Na_4_EDTA) solution at a pH between 12 and 13. After cleaning the scales formed in the tube, pump 2 pumps deionized water according to programed valve instructions. After scale removal, distilled water was injected for 10 min with a flow rate of 9.99 mL/min.

The scale rig is programed to complete four stages in each experiment as follows:

1. First blank: Just cationic and anionic solutions are pumped until scales are formed.
2. First scale: A series of programed SI concentrations are pumped for 1 h each or until scales are formed along with a cationic and an anionic solution.
3. Second scale: Step two is repeated to reconfirm and to avoid any misleading results.
4. Second blank: The same as the first bank test with no SI.

In general, the concentrations of SI were set to 100, 50, 20, 10, 5, 2, and 1 ppm for 1 h or until scales were formed in the microbore coil. The lower limit for the SI concentration, fail inhibition concentration (FIC), was set to a value where the differential pressure increased more than 0.5 bar (7 psi) (FIC should not be confused with MIC, which is the concentration that inhibits scale deposition). The inputs of the scale rig were programed and controlled by its software from a nearby computer. We prepared new brine solutions for each test, so it was not common to get exactly the same time values for different experiments due to the stochastic nature of the nucleation process. The compositions of two synthetic brines used in this work were based on the produced water from the Heidrun oilfield, North Sea, Norway, as tabulated in Table 2. We used a 50/50 volume mixture of formation water and synthetic seawater to afford the barite scale using all ions in Table 2, except bicarbonate ions. These brines were degassed by a vacuum pump for 15 min to avoid any gas bubble formation in flow tubes.

**Table 2:** Synthetic Brine Compositions for Scale Inhibition Testing of SIs

ion	Heidrun formation water (ppm)	seawater (ppm)	50/50 mixed brine (ppm)
Na^+^	19 500	10 900	15 200
Ca^2+^	1020	428	724
Mg^2+^	265	1368	816
K^+^	545	460	502
Ba^2+^	285	0	142
Sr^2+^	145	0	72
SO_4_^2–^	0	2960	1480
HCO_3_^–^	880	120	500

### Calcium Tolerance Test

The formation water in the petroleum reservoir has a high concentration of divalent ions such as calcium ions that can cause formation damage when they react with SIs.^4^ The incompatibility of SIs with brine compositions results in precipitation, which blocks the pores of formation rocks, leading to poor placement of SI during the squeeze treatment. Therefore, calcium tolerance tests are needed to check that SI matches the produced water composition without causing formation damage. It was reported that SI blended phosphonate groups can react with calcium ions affording SI–Ca complex precipitation.^28^ Generally, different concentrations of calcium ions and SIs were mixed in a synthetic seawater brine solution to check whether the formation of sparingly soluble calcium phosphonates occurred. In this test, four different concentrations of SI of 100, 1000, 10 000, and 50 000 ppm were dissolved in 20 mL of deionized water in 50 mL glass bottles. Then, 3.00% of NaCl and Ca^+2^ ions in doses from 10 to 10 000 ppm were added to corresponding bottles. The pH of the mixture solution was adjusted in the range of 4.0–4.5. The bottles were shaken well at room temperature until the solution became clear and then kept in an oven at 80 °C for 24 h. The mixture solutions were observed after 30 min, 1, 4, and 24 h. The haziness and/or precipitation of SI with Ca^+2^ ions in the synthetic seawater solution was determined and recorded by visual observations. This procedure was repeated for all inhibitors to check their calcium compatibilities.

### Hydrothermal Stability Test

The thermal aging test is needed to check whether SIs are stable (maintain performance) or unstable (lose performance) in the reservoir formation at elevated temperatures. It also helps to predict the squeeze lifetime of SIs. A 5 wt % SI solution in deionized water was added in a 50 mL pressure tube at pH 4.5. The pressure tube was connected to an air-free setup where the mixture was stirred under nitrogen gas for 1 h. Therefore, all residual oxygen was trapped by a vacuum pump until no more bubbles formed, and then the SI solution was aged at 130 °C for 7 days in acquired conditions. Thermally aged solutions were then evaluated against calcite and barite oilfield scales compared to nonaged inhibitors using the dynamic scale loop test.

## Results and Discussion

### Chemistry

Aromatic and aliphatic hydroxybisphosphonates were prepared in-house from low-cost and environmentally friendly carboxylic acid derivatives. All carboxylic acid groups in the structure backbone were phosphonated in the presence of phosphorus trichloride in methanesulfonic acid (MsOH). It was noted that there was no need to use a phosphorous acid as a cophosphonating agent, as reported by Grün et al.^31^ For aliphatic hydroxybisphosphonate SIs, we synthesized compounds with one or two bisphosphonate groups. A series of amino-end-capped alkane-hydroxybisphosphonates (with alkanes propane, butane, and pentane labeled as **SI-1**–**SI-3**, respectively) were synthesized and investigated as SIs for calcite and barite scales. Furthermore, from biscarboxylic acid starting materials, two new SIs containing two hydroxybisphosphonate groups (**SI-4** and **SI-5**) were synthesized for the first time and developed as antiscaling agents at oilfield conditions.^38^ For aromatic SIs, two biodegradable aromatic starting materials (e.g., benzoic acid and para-aminobenzoic acid) were functionalized with hydroxybisphosphonate groups using the same synthetic approach, giving **SI-6** and **SI-7**, respectively.

All chemical structures were characterized by ^1^H and ^13^P nuclear magnetic resonance (NMR) spectroscopy. ^31^P NMR is a common spectroscopic technique for elucidating organophosphorus compounds. For example, the ^1^H NMR spectra of **SI-1** showed a distinct triplet at δ 3.29 ppm for NH_2_–C**H**_2_ protons and a broad multiplet at δ 2.29–2.19 for C**H**_2_–COH(PO_3_H_2_)_2_ protons. In addition, the chemical shift of the phosphonic acid group (−PO_3_H_2_) of hydroxybisphosphonates **SI-1** showed a singlet signal at δ 17.00 ppm. It was also found that ^31^P NMR chemical shifts of all synthesized hydroxybisphosphonates were in the range of δ 14.00–18.00 ppm.

### High-Pressure Dynamic Tube-Blocking Test

The scale inhibition efficiencies of various hydroxybisphosphonate SIs were tested against calcite and barite scales by a high-pressure dynamic tube-blocking rig at 100 °C and 80 bars. All experimental results were collected from the first and repeat tests. Different concentrations of SIs of 100, 50, 20, 10, 5, 2, and 1 ppm were injected by pump 3 for 1 h or until the scale was formed at failed inhibition concentrations (FIC). Before injecting the SI into the scale rig, we adjusted the pH of all SIs in the range of 4–6 in 1000 ppm aqueous solution. It is very important to adjust the pH of SIs prepared in solution (pH = 4–6) to match the petroleum reservoir pH.

We have screened scale inhibition performances for all in-house-synthesized hydroxybisphosphonate SIs for calcite and barite scales in comparison with **HEDP**, **AEDP**, **and ATMP** (Tables 3 and 4). For the calcite scale, it was found that the FICs of **HEDP** were 1 ppm, after 35 and 36 min in the first and repeat tests, respectively. Interestingly, **HEDP** showed an excellent calcite scale inhibition performance compared to widely used commercial phosphonate SIs **DTPMP** and **ATMP**. **DTPMP** and **ATMP** afforded good inhibition performance with FIC values of 20 and 10 ppm, respectively.^33^ One reason for this weakest inhibition may be that **ATMP** and **DTPMP** are not highly compatible with calcium ions, as described previously in our published article.^28^ Also, **AEDP** gave a moderate calcite scale inhibition performance with an FIC of 20 ppm after 22 and 20 min in the first and second runs, respectively, as shown in Table 3.

**Table 3:** FIC Values for **HEDP**, **AEDP**, **ATMP**, and New Hydroxybisphosphonate SIs for Calcite Scale^b^

	calcite scale					
	first blank	first scale test		second scale test		second blank
SI (1000 ppm)^c^	time (min)	concn (ppm)	time (min)	concn (ppm)	time (min)	time (min)
**HEDP**	12	1	35	1	36	13
**AEDP**(28)	6	20	22	20	21	8
**ATMP**(33)	11	20	26	20	26	12
**SI-1**	17	2	26	2	26	17
**SI-2**	18	2	30	2	34	18
**SI-3**	12	2	19	2	19	12
**SI-4**	16	10	4	10	4	14
**SI-5**	16	10	40	10	35	14
**SI-6**	16	2	26	2	28	17
**SI-7**	15	2	18	2	19	14
**SI-1**^a^	17	2	31	2	32	19
**SI-2**^a^	14	10	24	10	20	14
**SI-3**^a^	16	1	42	1	43	15
**SI-4**^a^	14	10	56	10	56	15
**SI-5**^a^	14	20	46	20	50	14
**SI-6**^a^	12	5	10	5	12	13
**SI-7**^a^	15	5	25	5	30	16

a All synthesized SIs (**SI-1**–**SI-7**) were tested for calcite scale after thermal aging at 130 °C for 7 days.

b The accuracy for all numerical values was ±5 min.

c The pH of all SIs was adjusted in the range of 4–6 in 1000 ppm aqueous solution.

**Table 4:** FIC Values for **HEDP**, **AEDP**, **ATMP**, and New Hydroxybisphosphonate SIs for Barite Scale^b^

	barite scale					
	first blank	first scale test		second scale test		second blank
SI^c^ (1000 ppm)	time (min)	conc. (ppm)	time (min)	conc. (ppm)	time (min)	time (min)
**HEDP**	10	100	55	100	50	11
**AEDP**(28)	4	100	13	100	13	7
**ATMP**(33)	11	10	42	10	41	11
**SI-1**	10	100	20	100	20	10
**SI-2**	12	100	49	100	51	13
**SI-3**	10	100	25	100	21	11
**SI-4**	12	20	12	20	14	11
**SI-5**	10	20	28	20	23	11
**SI-6**	15	100	24	100	19	13
**SI-7**	10	100	15	100	14	10
**SI-4**^a^	11	20	7	20	10	10
**SI-5**^a^	11	20	11	20	9	11

a **SI-4** and **SI-5** were tested for barite scale after thermal aging at 130 °C for 7 days.

b The accuracy for all numerical values was ±5 min.

c The pH of all SIs was adjusted in the range of 4–6 in 1000 ppm aqueous solution.

**Figure 5:**
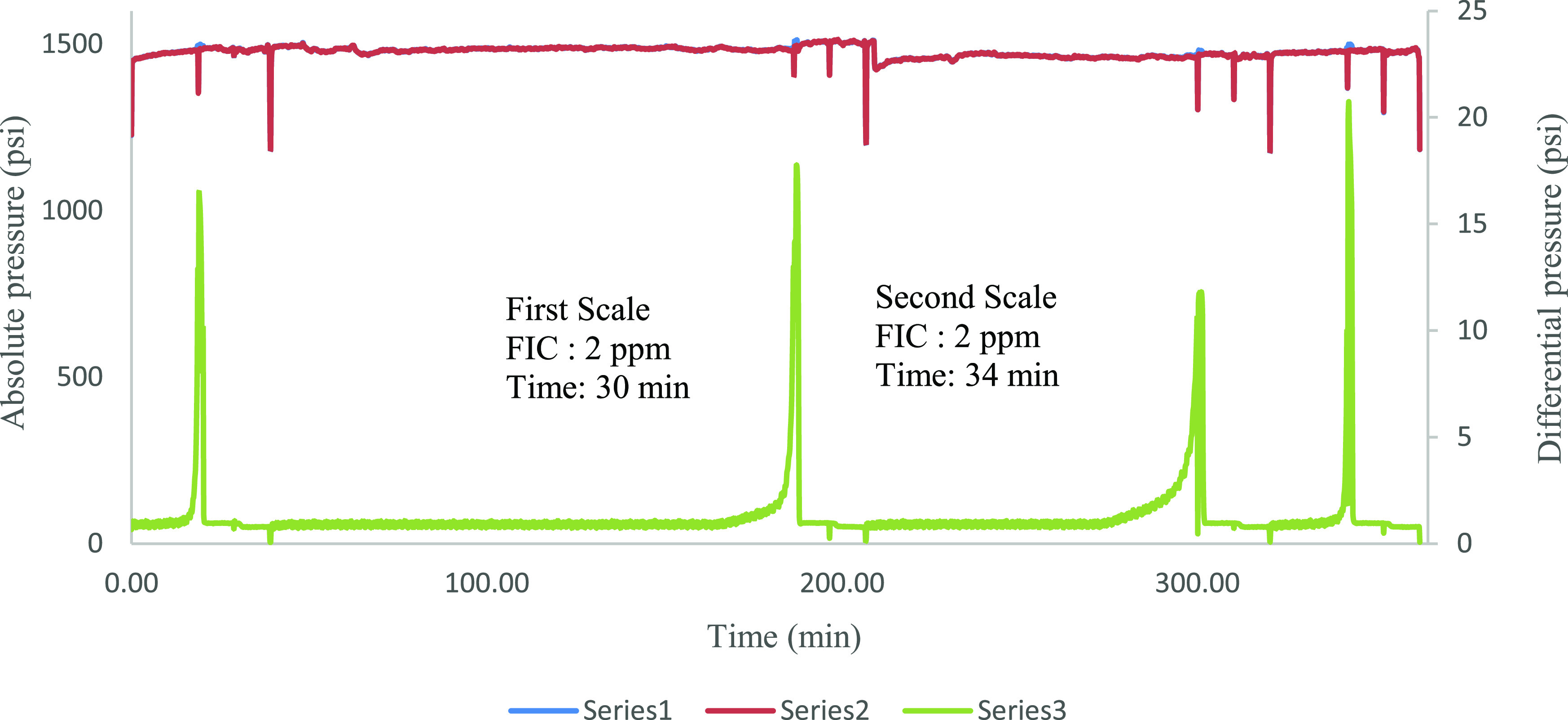
Pressure vs time graph of the calcite dynamic test results for **SI-2**.

For the aliphatic hydroxybisphosphonate SIs, all amino-end-capped alkane-hydroxybisphosphonates (with alkanes propane, butane, and pentane labeled as **SI-1**–**SI-3**, respectively) showed very good inhibition performance at 2 ppm for the calcite scale. Figure 5 shows the schematic graph of **SI-2** against calcite scaling in which the following four stages of the dynamic tube-blocking test can be seen: (1) a blank test with no inhibitor, (2) a test to detect the FIC, (3) a repeat FIC test, and (4) a repeat blank test. We pumped **SI-2** at 10, 5, 2, and 1 ppm for 1 h each. In the first stage, it was found that the scale formed after 18 min with no SI where the differential pressure increased above ca. 18 psi. The tubing was then cleaned using cleaning agents (Na_4_EDTA) and water, which brought the differential pressure back to 1 psi. In the second stage, the test started by pumping 10 ppm **SI-2**. No scale was detected at this concentration. The test kept running by pumping 5 and 2 ppm **SI-2**. After 30 min at 2 ppm, rapid scale deposition occurred. After the removal of scale by cleaning the coil with cleaning agents, the whole procedure was repeated to elucidate the repeatability of the tests (stage 3). It was found that the repeat FIC was 2 ppm after 34 min. In the fourth stage, the experiment was completed by another blank test with no SI. After 18 min, rapid scale formation happened, leading to good repeatability of the test. It was also observed that the distance between amino and hydroxybisphosphonate groups did not show a clear influence on the improved calcite scale inhibition performance.

Another class of aliphatic SIs with two hydroxybisphosphonate end groups has been developed. **SI-4** and **SI-5** were synthesized from biscarboxylic acid starting materials. These chemicals have different alkane lengths between the two hydroxybisphosphonate moieties. The high-pressure dynamic tube-blocking experimental results for **SI-4** and **SI-5** gave a moderate inhibition performance against the calcite scale. The FIC of **SI-4** (alkane, butane) was 10 ppm after 4 min for both runs. **SI-5** (alkane, hexane) failed at 10 ppm after 40 and 35 min for both runs, respectively. We speculate that the main reason for the weakest calcite scale inhibition performance of these chemicals is intolerance to calcium ions, leading to calcium–SI complex deposition.

As for the new aromatic SIs, two hydroxybisphosphonates based on aromatic compounds showed excellent calcite inhibition performance. **SI-6** (also known as fenidronic acid) indicated an FIC of 2 ppm after 26 and 28 min. In addition, the phosphonated *para*-aminobenzoic acid **SI-7** also afforded significant antiscaling activities with an FIC of 2 ppm after 18 and 19 min for both runs.

For the sulfate scale experiments, the FIC of the commercial product **HEDP** was 100 ppm after 55 and 50 min for both runs, respectively, as summarized in Table 4. We previously found that **AEDP** afforded a poor inhibition performance against the barite scale. The FIC was 100 ppm after 13 min for both runs, as tabulated in Table 4. In addition, all amino-end-capped alkane-hydroxybisphosphonates (with alkanes propane, butane, and pentane labeled as **SI-1**–**SI-3**, respectively) afforded poor inhibition performance for the barite scale. For example, the FIC of **SI-2** failed after 49 min for the first run and 51 min for the second run at 100 ppm (Table 4).

In contrast, our new hydroxybisphosphonate SIs (**SI-4** and **SI-5**) showed improved barite scale inhibition performance compared to the commercial product **HEDP** and the other synthesized hydroxybisphosphonate compounds. The FIC of **SI-4** was 20 ppm after 12 and 14 min for both tests, as presented in Table 4.

Moreover, the synthesized hydroxybisphosphonate SIs based on aromatic rings (**SI-6** and **SI-7**) exhibited a weak inhibition performance at 100 ppm for the barite scale. The FICs of **SI-6** gave rapid precipitation after 24 min in the first test and 19 min in the second test at 100 ppm, as shown in Table 4.

We suggest that the significant inhibition performances of **SI-4** and **SI-5** for the barite scale may be related to extra bisphosphonate moieties on the SI backbone. As investigated in our previously published articles, the chemicals with several phosphonate groups show improved barite scale inhibition performance.^28,33^ It was reported that the products with more than one phosphonate groups increased the active sites for phosphonate binding and inhibition of the barite scale. This phenomenon was illustrated by the graphical molecular modeling package Chem-X7.^39^

We can summarize, on the basis of the above results, that most of the new synthesized hydroxybisphosphonate SIs gave an excellent inhibition performance against the calcium carbonate scale. Furthermore, bisphosphonate SIs based on hydroxyl groups gave a better calcite scale inhibition performance than the bisphosphonate SIs incorporating amino groups. We speculate that the hydroxyl group plays a key role in the oilfield scale inhibition performance. In addition, aliphatic and aromatic hydroxybisphosphonate SIs showed weak inhibition activities for the barium sulfate scale. However, the inhibition performance of these classes of scale inhibitors can be improved by adding extra bisphosphonate groups in their backbone structures. For example, **SI-4** and **SI-5** containing two hydroxybisphosphonate groups showed reasonable scale inhibition performances compared to other SIs incorporating only one hydroxybisphosphonate group. Overall, the repeatability of the scale inhibition test method afforded similar results at the same test conditions. This confirmed that the high-pressure dynamic tube-blocking test is a suitable technique for measuring the accuracy and precision of obtained results.

### Thermal Stability Test

Organophosphororous SIs are widely used in topside and downhole squeeze treatments. There is a clear need for developing improved SIs for use in downhole squeeze applications under harsh conditions such as high temperatures. Laboratory thermal aging tests were performed for all hydroxybisphosphonate SIs for the calcite scale. In addition, we carried out long-term thermal aging tests for the best SIs for the barite scale. These chemicals were thermally aged at 130 °C for 7 days under the protection of nitrogen gas. Tables 3 and 4 show the scale inhibition performances of selected thermally aged SIs.

**Figure 6:**
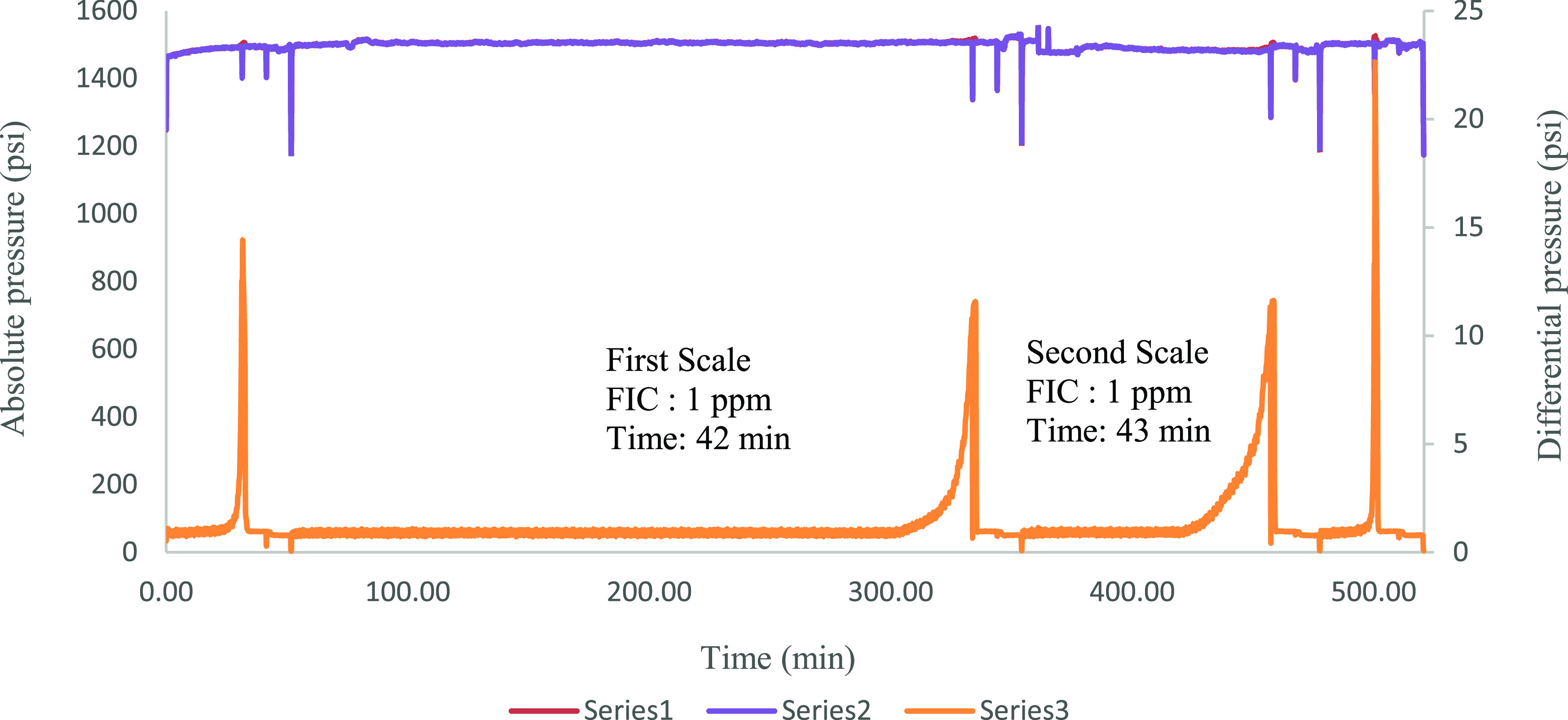
FIC and time values for high-pressure dynamic tube-blocking experiments of **SI-3** after thermal aging at 130 °C for the calcite scale.

For the calcite scale, the FIC of **SI-1** was 2 ppm after 31–32 min after each run. **SI-2** lost its inhibition performance after thermal aging at 130 °C, going from an FIC of 2 to 10 ppm. Interestingly, **SI-3** showed improved calcite inhibition performance compared to the unaged sample. The FIC was changed from 2 to 1 ppm, as presented in Figure 6 and Table 3. We have repeated the whole test to elucidate the reproducibility of the tests and obtained similar results. Furthermore, **SI-4** revealed improved thermal stability activities with an FIC of 10 ppm after 56 min for both experiments. We do not currently have a real explanation for the improved calcite inhibition performance of aged **SI-3** and **SI-4**.

The high-pressure dynamic tube-blocking experiments showed that **SI-5** lost its performance, dropping from 10 to 20 ppm. In addition, aromatic hydroxybisphosphonates **SI-6** and **SI-7** were not thermally stable at 130 °C, giving an FIC of 5 ppm for both runs. Compared to the unaged inhibition performance results for the barite scale, **SI-4** and **SI-5** gave the same inhibition performances after thermal aging at 130 °C for 1 week. For example, the FIC of **SI-4** was 20 ppm after 7–10 min in the first and second runs as stated in Table 4.

### Calcium Compatibility Test

As stated earlier, organophosphorus compounds are well-known SIs in the upstream oil and gas industry. However, most of these chemicals have at least one drawback, such as intolerance to high calcium ion concentrations, leading to SI–Ca^+2^ complex precipitation. To evaluate calcium ion tolerance toward the synthesized SIs, a series of compatibility tests were carried out at 80 °C in the presence of 30 000 ppm NaCl. The calcium ion concentrations were in the range of 10–10 000 ppm, whereas the SI concentrations were changed from 100 to 50 000 ppm. **HEDP** showed good calcium tolerance at all SI concentrations for 10 ppm calcium ions. In addition, **HEDP** showed poor to moderate calcium compatibility performance at 1000–50 000 ppm SI concentrations for 100–10 000 ppm calcium ions. For example, **HEDP** showed good calcium compatibility at 100 and 1000 ppm SI and 10 000 ppm calcium ions over the 24 h test period. However, hazy solutions and precipitates were observed in the presence of 10 000–50 000 ppm SI concentrations and 1000 ppm calcium ions under the same conditions.

It was also found that all amino-end-capped alkane-hydroxybisphosphonates (**S1-1**, **SI-2**, and **SI-3**) exhibited good calcium compatibility at all SI concentrations and 10 ppm calcium ions. However, these chemicals showed moderate calcium compatibility activities at 100–10 000 ppm calcium ions. The calcium compatibility results of **SI-1** are summarized in Tables 5^6^–7.

**Table 5:** Tolerance Tests in 100 ppm Ca^2+^ and 30 000 ppm (3.0 wt %) NaCl for **SI-1**

	appearance				
dose (ppm)	at mixing	30 min	1 h	4 h	24 h
100	clear	clear	clear	clear	clear
1000	clear	clear	clear	clear	clear
10 000	clear	clear	haze	haze	haze
50 000	precipitated	precipitated	precipitated	precipitated	precipitated

**Table 6:** Tolerance Tests at 1000 ppm Ca^2+^ and 30 000 ppm (3.0 wt %) NaCl for **SI-1**

	appearance				
dose (ppm)	after mixing	30 min	1 h	4 h	24 h
100	clear	clear	clear	clear	clear
1000	clear	clear	clear	clear	clear
10 000	precipitated	precipitated	precipitated	precipitated	precipitated
50 000	precipitated	precipitated	precipitated	precipitated	precipitated

**Table 7:** Tolerance Tests in 10 000 ppm Ca^2+^ and 30 000 ppm (3.0 wt %) NaCl for **SI-1**

	appearance				
dose (ppm)	after mixing	30 min	1 h	4 h	24 h
100	clear	clear	clear	clear	clear
1000	clear	clear	clear	clear	clear
10 000	precipitated	precipitated	precipitated	precipitated	precipitated
50 000	precipitated	precipitated	precipitated	precipitated	precipitated

Furthermore, **SI-4** and **SI-5** showed very good calcium compatibility at all SI concentrations and 10–100 ppm calcium ions. For 1000 and 10 000 ppm calcium ions, the compatibility was worse due to precipitate formation at most of the SI concentrations. As for aromatic hydroxybisphosphonate SIs, **SI-6** gave an excellent tolerance performance at all concentrations of calcium ions throughout the 24 h test period. In addition, **SI-7** showed poor to moderate calcium compatibility activities. We assume that the amino group in the backbone structure of hydroxybisphosphonate SIs based on aromatic rings has an essential role in decreasing calcium compatibility performance.

## Conclusions

The low toxicity of bisphosphonates encouraged us to design and synthesize a series of aliphatic and aromatic hydroxybisphosphonates (labeled as **SI-1**–**SI-7**) as oilfield SIs. Initially, it was found that the commercial 1-hydroxyethylidene bisphosphonic acid (**HEDP**) gave a better calcite inhibition performance compared to our previously synthesized 1-aminoethylidene bisphosphonic acid (**AEDP**) under the same test conditions. This shows that the hydroxyl group improves the binding affinity of the bisphosphonate derivatives. Table 8 summarizes the qualitative experimental results for SI performance (barite and calcite), calcium tolerance, and thermal stability at 130 °C for 7 days.

**Table 8:** Qualitative Summary of Results for New Oilfield SIs

SI	calcite SI	barite SI	thermal aging	calcium tolerance
**SI-1**	very good	poor	excellent	fair
**SI-2**	very good	poor	fair	fair
**SI-3**	very good	poor	excellent	fair
**SI-4**	good	fair	excellent	fair
**SI-5**	good	fair	very good	fair
**SI-6**	very good	poor	fair	excellent
**SI-7**	very good	poor	fair	fair

As shown in the above table, no chemical matched all test categories, showing the challenge in designing new SIs with all of the features required for the oilfield application. For example, amino-end-capped alkane-hydroxybisphosphonates (with alkanes propane, butane, and pentane labeled as **SI-1**–**SI-3**, respectively) gave an outstanding inhibition performance against calcite scaling. In addition, **SI-1** and **SI-3** are thermally stable at 130 °C for 7 days under anaerobic conditions. However, these chemicals showed poor calcium compatibility with calcium ions up to 1000 ppm. Furthermore, they stood out as poor barite SIs under the test conditions. Hydroxybisphosphonate-based aromatic rings (**SI-6** and **SI-7**) exhibited a very good calcite scale inhibition performance. Interestingly, **SI-6** gave an excellent calcium compatibility activity at all inhibitor concentrations for 10, 100, 1000, and 10 000 ppm calcium ions. It was also found that hydroxybisphosphonate SIs containing two bisphosphonate groups (**SI-4** and **SI-5**) afforded the best barite scale inhibition performance compared to other synthesized hydroxybisphosphonates and commercial product HEDP. This indicates that the barite inhibition performance of these SIs can be improved by adding extra bisphosphonate groups on their backbone structures.

Overall, the results show that low-toxicity hydroxybisphosphonates can be proposed as potential candidates for scale inhibitors against the calcite scale for topside and downhole applications in the presence of low calcium ion concentrations. We plan to improve the calcium compatibility of hydroxybisphosphonate SIs by capping other functional groups, such as sulfonate.

## Supporting Information Available

Synthesis of new BPs; Table S1, chemical structure and spectral data of aliphatic and aromatic hydroxybisphosphonate SIs; and Tables S2–S17, compatibility tests for hydroxybisphosphonate SIs (PDF)
