# Supplementary material for: Synthesis and Antiscaling Evaluation of Novel Hydroxybisphosphonates for Oilfield Applications
Source: ACS Omega. 2021 Feb 25;6(9):6488–97. doi: 10.1021/acsomega.1c00379 (PMC7948439; doi:10.1021/acsomega.1c00379)
Supplement: Supplementary file 1 — ao1c00379_si_001.pdf [file ao1c00379_si_001.pdf]

Supporting Information

FOR

Synthesis and Antiscaling Evaluation of Novel Hydroxybisphosphonates for Oilfield Applications

Authors: Mohamed F. Mady[a,b*], Abdur Rehman[a], Malcolm A. Kelland[a]

[a]Department of Chemistry, Bioscience and Environmental Engineering, Faculty of Science and Technology, University of Stavanger, N-4036 Stavanger, Norway.

[b]Department of Green Chemistry, National Research Centre, Dokki, Cairo 12622, Egypt

Contents:

Synthesis of new BPs	S2-S3
Table S1. Chemical structure and spectra data of aliphatic and aromatic hydroxybisphosphonate SIs.	S3-S4
Tables S2-S17: Compatibility tests for hydroxybisphosphonate SIs	S5-S10

Synthesis of new BPs:

(*1,4-dihydroxybutane-1,1,4,4-tetrayl)tetrakisphosphonic acid*= (**SI-4**): In a two-neck round-bottom flask under nitrogen, 3.0 g (25.40 mmol) of succinic acid was added into 21.97 mL (228.62 mmol) of methanesulfonic acid under stirring at room temperature. Then, 22.32 g (162.53 mmol) of phosphorous trichloride was added dropwise for ca. 30 min and allowed to heat stepwise from room temperature to 75°C. The reaction mixture was stirred for 48 h at 75°C. After that, the mixture was cooled to room temperature and quenched carefully with 39.12 mL of deionized H_2O. The reaction solution was then heated up and refluxed at 105 °C for 16 h. Next, the pH of the flask contents was adjusted to 1.8 by adding a 50% aqueous solution of NaOH, and then 127 mL of methanol was added into the mixture and followed by stirring for 45 mins at room temperature. The crude product was collected by filtration and dissolved in 10 mL of hot water. Furthermore, 50 mL of methanol was added dropwise into the flask, providing an off-white solid. The phosphonated succinic acid (**SI-4**) was isolated as a pure product in 43% yield (Table S1).

((4-aminophenyl)(hydroxy)methylene)bis(phosphonic acid) = (**SI-7**): In a two-neck round-bottom flask under nitrogen, 3.0 g (21.87 mmol) of succinic acid was added into 9.46 mL (98.43 mmol) of methanesulfonic acid under stirring at room temperature. Then, 9.61 g (70.00 mmol) of phosphorous trichloride was added dropwise for ca. 30 min and allowed to heat stepwise from room temperature to 75°C. The reaction mixture was stirred for 48 h at 75°C. After that, the mixture was cooled to room temperature and quenched carefully with 39.12 mL of deionized H_2O. The reaction solution was then heated up and refluxed at 105°C for 16 h. Next, the pH of the flask contents was adjusted to 1.8 by adding a 50% aqueous solution of NaOH, and then 127 mL of methanol was added into the mixture, followed by stirring for 45 min at room temperature. The crude product was collected by filtration and dissolved in 10 mL of hot

water. Furthermore, 50 mL of methanol was added dropwise into the flask, providing an off-white solid. **SI-7** was isolated as a pure product in 65% yield (Table S1).

Table S1. Chemical structure and spectra data of hydroxybisphosphonate SIs.

Compound	Spectral data
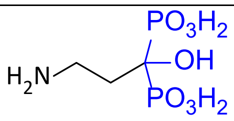 (SI-1)	3-amino-1-hydroxypropane-1,1-bisphosphonic acid = Pamidronic acid = (**SI-1**): ^{1}H NMR (D_2O, 400 MHz) δ ppm: 3.29 (t, 2H, NH_2-C**<u>H</u>**$_2$), 2.29-2.19 (m, 2H, C**<u>H</u>**$_2$-COH$(PO_3H_2)_2$; ^{31}P NMR (D_2O, 162.00 MHz) δ 17.00 ppm. Yield: 67%
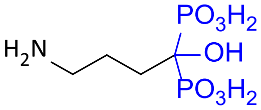 (SI-2)	4-amino-1-hydroxybutane-1,1-bisphosphonic acid = Alendronic acid = (**SI-2**):<br><br>^{1}H NMR (D_2O, 400 MHz) δ ppm: 2.95 (t, 2H, NH_2-C**<u>H</u>**$_2$), 2.01-1.87 (m, 4H, NH_2-CH_2-C**<u>H</u>**$_2$+ C**<u>H</u>**$_2$-COH$(PO_3H_2)_2$; ^{31}P NMR (D_2O, 162.00 MHz) δ 18.49 ppm. Yield: 69%
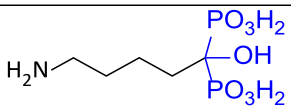 (SI-3)	5-amino-1-hydroxypentane-1,1-bisphosphonic acid = (**SI-3**): ^{1}H NMR (D_2O, 400 MHz) δ ppm: 2.94 (t, 2H, NH_2-C**<u>H</u>**$_2$), 1.92-1.82 (m, 2H, C**<u>H</u>**$_2$-COH$(PO_3H_2)_2$, 1.64-155 (m, 4H, NH_2-CH_2-C**<u>H</u>**$_2$- C**<u>H</u>**$_2$); ^{31}P NMR (D_2O, 162.00 MHz) δ 18.24 ppm. Yield: 64%
	(1,4-dihydroxybutane-1,1,4,4-tetrayl)tetrakisphosphonic acid = (**SI-4**): ^{1}H NMR (D_2O, 400 MHz) δ ppm: 2.14 (s, 4H,

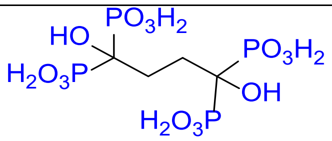 (SI-4)	-C**H**$_2$-C**H**$_2$-); ^{31}P NMR (D_2O, 162.00 MHz) δ 17.69 ppm. Yield: 43%
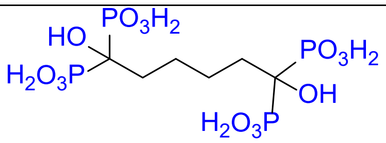 (SI-5)	(1,6-dihydroxyhexane-1,1,6,6-tetrayl)tetrakisphosphonic acid = (**SI-5**)*:* ^{1}H NMR (D_2O, 400 MHz) δ ppm: 1.95-1.80 (m, br, 4H, -C**H**$_2$-C**H**$_2$-), 1.56-1.47 (m, br, 4H, -C**H**$_2$-C**H**$_2$-); ^{31}P NMR (D_2O, 162.00 MHz) δ 18.66 ppm. Yield: 62%
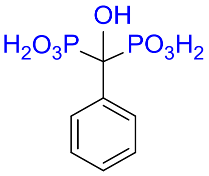 (SI-6)	Hydroxyphenylmethylene-1,1-bis phosphonic acid= Fenidronic acid = (**SI-6**)*:* ^{1}H NMR (D_2O, 400 MHz) δ ppm: 7.71-7.24 (m, br, 5H, -Aromatic); ^{31}P NMR (D_2O, 162.00 MHz) δ 15.60 ppm. Yield: 60%
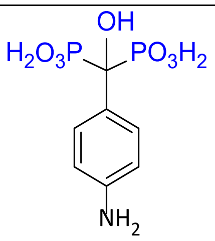 (SI-7)	((4-aminophenyl)(hydroxy)methylene)bisphosphonic acid = (**SI-7**)*:* ^{1}H NMR (D_2O, 400 MHz) δ ppm: 7.84-7.28 (m, br, 4H, -Aromatic); ^{31}P NMR (D_2O, 162.00 MHz) δ 14.87 ppm. Yield: 65%

Table S2. Tolerance tests in 1000 ppm of Ca^{+2} and 30000 ppm (3.0 wt.%) NaCl for **HEDP**.

Dose (ppm)	Appearance				
	at mixing	30 mins	1 hour	4 hours	24 hours
100	clear	clear	clear	clear	clear
1000	clear	clear	clear	clear	clear
10000	haze	haze	haze	haze	precipitated
50000	haze	haze	haze	haze	precipitated

Table S3. Tolerance tests in 1000 ppm of Ca^{+2} and 30000 ppm (3.0 wt.%) NaCl for **SI-2**.

Dose (ppm)	appearance				
	at mixing	30 mins	1 hour	4 hours	24 hours
100	clear	clear	clear	clear	clear
1000	clear	haze	precipitated	precipitated	precipitated
10,000	clear	precipitated	precipitated	precipitated	precipitated
50,000	precipitated	precipitated	precipitated	precipitated	precipitated

Table S4. Tolerance tests in 10000 ppm of Ca^{+2} and 30000 ppm (3.0 wt.%) NaCl for **SI-2**.

Dose (ppm)	Appearance				
	after mixing	30 mins	1 hour	4 hours	24 hours
100	clear	clear	clear	clear	clear
1000	precipitated	precipitated	precipitated	precipitated	precipitated
10000	precipitated	precipitated	precipitated	precipitated	precipitated
50000	precipitated	precipitated	precipitated	precipitated	precipitated

Table S5. Tolerance tests in 100 ppm of Ca^{+2} and 30000 ppm (3.0 wt.%) NaCl for **SI-3**.

Dose (ppm)	Appearance				
	after mixing	30 mins	1 hour	4 hours	24 hours
100	clear	clear	clear	clear	clear
1000	clear	clear	clear	clear	precipitated
10,000	clear	haze	haze	precipitated	precipitated
50,000	precipitated	precipitated	precipitated	precipitated	precipitated

Table S6. Tolerance tests in 1000 ppm of Ca^{+2} and 30000 ppm (3.0 wt.%) NaCl for **SI-3**.

Dose (ppm)	Appearance				
	after mixing	30 mins	1 hour	4 hours	24 hours
100	clear	clear	clear	clear	clear
1000	clear	clear	clear	clear	precipitated
10,000	clear	haze	haze	precipitated	precipitated
50,000	precipitated	precipitated	precipitated	precipitated	precipitated

Table S7. Tolerance tests in 10000 ppm of Ca^{+2} and 30000 ppm (3.0 wt.%) NaCl for **SI-3**.

Dose (ppm)	Appearance				
	after mixing	30 mins	1 hour	4 hours	24 hours
100	clear	clear	clear	clear	clear
1000	clear	clear	clear	clear	precipitated
10,000	clear	haze	haze	precipitated	precipitated
50,000	precipitated	precipitated	precipitated	precipitated	precipitated

Table S8. Tolerance tests in 100 ppm of Ca^{+2} and 30000 ppm (3.0 wt.%) NaCl for **SI-4**.

Dose (ppm)	Appearance				
	after mixing	30 mins	1 hour	4 hours	24 hours
100	clear	clear	clear	clear	clear
1000	clear	clear	clear	clear	clear
10,000	clear	clear	clear	clear	clear
50,000	clear	clear	clear	clear	clear

Table S09. Tolerance tests in 1000 ppm of Ca^{+2} and 30000 ppm (3.0 wt.%) NaCl for **SI-4**.

Dose (ppm)	Appearance				
	after mixing	30 mins	1 hour	4 hours	24 hours
100	precipitated	precipitated	precipitated	precipitated	precipitated
1000	precipitated	precipitated	precipitated	precipitated	precipitated
10,000	precipitated	precipitated	precipitated	precipitated	precipitated
50,000	precipitated	precipitated	precipitated	precipitated	precipitated

Table S10. Tolerance tests in 10000 ppm of Ca^{+2} and 30000 ppm (3.0 wt.%) NaCl for **SI-4**.

Dose (ppm)	Appearance				
	after mixing	30 mins	1 hour	4 hours	24 hours
100	precipitated	precipitated	precipitated	precipitated	precipitated
1000	precipitated	precipitated	precipitated	precipitated	precipitated
10,000	precipitated	precipitated	precipitated	precipitated	precipitated
50,000	precipitated	precipitated	precipitated	precipitated	precipitated

Table S11. Tolerance tests in 100 ppm of Ca^{+2} and 30000 ppm (3.0 wt.%) NaCl for **SI-5.**

Dose (ppm)	Appearance				
	after mixing	30 mins	1 hour	4 hours	24 hours
100	clear	clear	clear	clear	clear
1000	clear	clear	clear	clear	clear
10,000	clear	clear	clear	clear	clear
50,000	clear	clear	clear	clear	clear

Table S12. Tolerance tests in 1000 ppm of Ca^{+2} and 30000 ppm (3.0 wt.%) NaCl for **SI-5.**

Dose (ppm)	Appearance				
	after mixing	30 mins	1 hour	4 hours	24 hours
100	clear	clear	clear	clear	clear
1000	clear	clear	clear	clear	clear
10,000	clear	haze	haze	precipitated	precipitated
50,000	clear	haze	haze	precipitated	precipitated

Table S13. Tolerance tests in 10000 ppm of Ca^{+2} and 30000 ppm (3.0 wt.%) NaCl for **SI-5.**

Dose (ppm)	Appearance				
	after mixing	30 mins	1 hour	4 hours	24 hours
100	precipitated	precipitated	precipitated	precipitated	precipitated
1000	precipitated	precipitated	precipitated	precipitated	precipitated
10,000	precipitated	precipitated	precipitated	precipitated	precipitated
50,000	precipitated	precipitated	precipitated	precipitated	precipitated

Table S14**.** Tolerance tests in 10000 ppm of Ca^{2+} and 30 000 ppm (3.0 wt %) of NaCl for **SI-6**.

Dose (ppm)	Appearance				
	after mixing	30 mins	1 hour	4 hours	24 hours
100	clear	clear	clear	clear	clear
1000	clear	clear	clear	clear	clear
10000	clear	clear	clear	clear	clear
50000	clear	clear	clear	clear	clear

Table S15. Tolerance tests in 100 ppm of Ca^{+2} and 30000 ppm (3.0 wt.%) NaCl for **SI-7.**

Dose (ppm)	Appearance				
	after mixing	30 mins	1 hour	4 hours	24 hours
100	clear	clear	clear	clear	clear
1000	clear	clear	clear	clear	clear
10,000	clear	haze	haze	precipitated	precipitated
50,000	clear	haze	haze	precipitated	precipitated

Table S16. Tolerance tests in 1000 ppm of Ca^{+2} and 30000 ppm (3.0 wt.%) NaCl for **SI-7.**

Dose (ppm)	Appearance				
	after mixing	30 mins	1 hour	4 hours	24 hours
100	clear	clear	clear	precipitated	precipitated
1000	clear	clear	clear	precipitated	precipitated
10,000	clear	precipitated	precipitated	precipitated	precipitated
50,000	clear	precipitated	precipitated	precipitated	precipitated

Table S17. Tolerance tests in 10000 ppm of Ca^{+2} and 30000 ppm (3.0 wt.%) NaCl for **SI-7.**

Dose (ppm)	Appearance				
	after mixing	30 mins	1 hour	4 hours	24 hours
100	clear	clear	clear	precipitated	precipitated
1000	clear	precipitated	precipitated	precipitated	precipitated
10,000	clear	precipitated	precipitated	precipitated	precipitated
50,000	clear	precipitated	precipitated	precipitated	precipitated
